# Inspecting the factors of individualizing and binding moral value orientation in the Moral Foundations Questionnaire-2 for validation—a re-analysis of data from pre-service teachers in Ghana

**DOI:** 10.3389/fpsyg.2025.1665536

**Published:** 2025-11-26

**Authors:** Steffen Wild, Daniel Fobi, Michélle Möhring

**Affiliations:** 1 Department of Rehabilitation Sciences, Technical University Dortmund, Dortmund, Germany; 2 Department of Special Education, University of Education Winneba, Winneba, Ghana

**Keywords:** factor structure, Ghana, binding, individualizing, MFQ-2, moral foundations, non-WEIRD country

## Abstract

**Introduction:**

*Moral foundation theory* postulates two higher-order moral value orientations: individualizing and binding. In the measurement instrument of the *Moral Foundations Questionnaire-2* (MFQ-2), 36 items cover the dimensions of authority, care, equality, loyalty, proportionality, and purity, which contribute to people’s individualizing and binding moral value orientation. So far, less research exists for the validation of the moral value structure in so-called non-WEIRD (western, educated, industrialized, rich, and democratic) countries like Ghana. Thus, the question arises: what is the empirical structure of moral value orientations in Ghana, and is it possible to identify the factors of individualizing and binding moral value orientations in line with the theoretical framework?.

**Methods:**

We re-analyse data from 1,049 pre-service teachers at a university in Ghana that were gathered using a cross-sectional design and convenience sampling.

**Results:**

Our re-analyses provide first hints of construct validity as well as criterion validity with the criteria of gender and religiosity. The abovementioned six underlying dimensions could be seen as first-order factors. The assumption of individualizing and binding moral value orientations as second-order factors in the MFQ-2 is weakly supported.

**Discussion:**

Findings are reflected upon and discussed in terms of limitations. Further investigations in other populations of non-WEIRD countries are deemed necessary to evaluate the instrument for robustness.

## Highlights

- The Moral Foundations Questionnaire-2 (MFQ-2) has undergone a further validation.
- Data from a non-WEIRD country, Ghana, is re-analyzed, where the instrument has never been used before.
- More than 1,000 participants, pre-service teachers, are included in the sample.
- First hints for construct validity and criterion validity are given.
- We did not find strong support for the assumption of individualizing and binding moral value orientation as second-order factors in our sample.
- The six moral foundations could be seen as robust first-order factors in our analyses.

## Introduction

*Moral foundations theory* (MFT; Haidt and Joseph, 2004) proposes that moral judgments emerge from a dynamic interplay between innate predispositions (nativism), culturally transmitted norms (cultural learning), and the coexistence of diverse moral outlooks (moral pluralism). Recognizing these assumptions is crucial for understanding both the universality and variability of moral judgments across societies, thereby enriching contemporary debates in moral psychology.

Empirical research now investigates human morality systematically after centuries of speculation (Dogruyol et al., 2024). Currently, a new instrument named *Moral Foundations Questionnaire–2* (MFQ-2) by Atari et al. (2023) is suggested and discussed, which is rooted in MFT. Following Goenka and Thomas (2024), it is emphasized on the one hand that the moral values of care and fairness build up people’s individualizing moral value orientation with the function of protecting the equality and welfare of individuals by providing individual rights in society. On the other hand, the moral values of authority, loyalty, proportionality, and purity build up people’s binding moral value orientation with the function of connecting people in a larger group and maintaining the group’s welfare.

The structural analysis of the MFQ-2 is seen as essential (Wormley et al., 2023) because, to date, no well-fitting model of the MFQ exists (Zakharin and Bates, 2021). Another challenge is that the validity of MFT in non-WEIRD (Western, educated, industrialized, rich, and democratic; Henrich et al., 2010) countries is less researched (Dogruyol et al., 2024), and to our best knowledge, no research has been done in Ghana using the proposed measuring instrument MFQ-2. Nilsson (2023) summarizes that evidence of measurement invariance in morality instruments is scarce. Furthermore, pre-service teachers are an important group because of their work task as teachers: They have a selective function of students for society, such as giving grades that regulate access to the next levels of the education system or occupational positions, and transmitting knowledge, skills, and values to the next generation (Neugebauer, 2019).

Winkelkotte et al. (2025) use *item response theory* (IRT) to initially investigate the validity of the six moral foundations: care, fairness, authority, loyalty, proportionality, and purity, measured with the MFQ-2 in Ghana. However, Winkelkotte et al. (2025) did not analyze the data regarding the background of people’s individualizing or binding moral value orientation at a higher level. Consequently, we re-analyze the data based on the initial results by Winkelkotte et al. (2025) to elaborate on the higher-order factor structure of the MFQ-2, which is seen as an analysis of factorial validity. In parallel, we analyze the instrument’s criterion and construct validity in our study. Accordingly, we investigate the validity of the MFQ-2 in Ghana.

## Moral foundations theory

Scholars emphasize, across time and academic disciplines, that morality is rooted in social relations (Kouchaki et al., 2013). These assumptions are also reflected in the definition of morality by Haidt and Kesebir (2010; p. 800), who state that “moral systems are interlocking sets of values, virtues, norms, practices, identities, institutions, technologies, and evolved psychological mechanisms that work together to suppress or regulate selfishness and make social life possible.” As a consequence, morality regulates social processes within groups to keep them together and, in parallel, ensures the wellbeing of individuals in those groups. The framework of the MFT gives one possible explanation for the underlying concepts in the development of morality. The MFT framework is based on the following assumptions: (1) intuitionism, (2) nativism, (3) cultural learning, and (4) moral pluralism (Graham et al., 2013).

First, *intuitionism*, based on the *Social Intuitionist Model* (Haidt, 2001), proclaims that “intuitions come first, strategic reasoning second” (Haidt, 2012, p. 3). Consequently, moral decisions are grounded more in (unconscious) emotional intuitions than in (conscious) cognitional decisions.

The second assumption of *nativism* in moral development follows two ideas: On the one hand, there is the concept of reductionism (behavior is genetically hardwired in an organism and can be performed in response to a cue without prior experience), and on the other hand, the approach of constructivism (behavior is learned in the course of life). In other words, it is assumed that an initial ‘draft’ of morality is provided by the genetic disposition of a person, which, in the sense of a cultural learning process, is then further developed and adapted through differentiations and experiences (Graham et al., 2013; Graham et al., 2018).

The third assumption of *cultural learning* must be seen in the context of the previous ideas of MFT. It is stated that there must exist some moral foundations that influence human morality. It is assumed that the foundations developed evolutionarily in humankind and are innate in every person. But it must be acknowledged that experiences in people’s lives, as well as their cultural background, are factors influencing these foundations (Graham et al., 2013; Haidt and Joseph, 2004).

The fourth assumption of MFT is *moral pluralism*, meaning that there is no moral monism, as in the theory of Kohlberg’s (1969) stages of moral development, for example. The MFT is grounded on the assumption that a pluralism of moral values exists. Haidt and Kesebir (2010; p. 800) summarize that “there are multiple incompatible but morally defensible ways of organizing a society.” To date, a distinction is made between the moral foundations of care, fairness, authority, loyalty, proportionality, and purity. Each moral foundation links specific emotional reactions and behavioral intentions (e.g., Graham et al., 2009).

## Notes on validity of MFQ

We begin with a brief introduction to the foundations of the MFQ measurement instrument. In an early suggested measurement instrument by Graham et al. (2011) named MFQ-1, five moral domains or moral foundations (harm/care, fairness/reciprocity, ingroup/loyalty, authority/respect, and purity/sanctity) were suggested. Meanwhile, an adjusted version of the instrument has been elaborated by Atari et al. (2023) that is named MFQ-2. This new MFQ-2 integrates the six foundations: care, equality, proportionality, loyalty, authority, and purity. At a higher level, a distinction is made between individualizing moral foundations—here the dimensions of harm and fairness (MFQ-1)/care and equality (MFQ-2) are summarized—and binding moral foundations—here the dimensions of ingroup, authority, and purity (MFQ-1)/proportionality, loyalty, authority, and purity (MFQ-2) are summarized.

The MFT has primarily been studied in WEIRD contexts (Atari et al., 2020) and, according to Winkelkotte (2023), has not yet been systematically explored in Ghana—as an example of a non-WEIRD country. However, when considering African ethics in general, and Akan ethics in particular—the Akan constitute the largest ethnic group in Ghana (Ghana Statistical Service, 2021)—clear parallels to MFT emerge. African moral thought is commonly understood as communitarian (Appiah-Sekyere, 2016; Metz, 2017; Murove, 2020), emphasizing self-realization through social relationships. This is reflected in wellknown proverbs such as “A person is a person through other persons” (Metz, 2017, p. 63). Similar to the MFT-postulated binding foundations, Akan ethics stress the individual’s integration into the community, extending obligations not only to the living but also to ancestors (Murove, 2020).

The Akan worldview highlights loyalty and care as moral imperatives, expressed in solidarity, cooperation, and nurturing within the community (Metz, 2017; Murove, 2020). Moreover, Akan culture, like much of African society, is profoundly shaped by religiosity and belief in supernatural forces (Appiah-Sekyere, 2016), which resonates with a high emphasis on the MFT foundation of purity/sanctity. Yet, moral values are not solely derived from divine sources; rather, they rest on humanistic concerns for communal welfare, encompassing virtues such as generosity, honesty, justice, respect, hospitality, and compassion (Gyekye and Nodelman, 2010; Metz, 2017). Respect for authority is central as well, extending to parents, elders, and traditional chiefs (Appiah-Sekyere, 2016)—a clear parallel to MFT’s foundation of authority.

Finally, the Akan emphasize the value of life itself. Actions that preserve life are considered morally good, while those that destroy life—such as abortion or suicide—are strongly condemned (Appiah-Sekyere, 2016; Metz, 2017). Thus, akin to MFT’s individualizing foundations, Akan ethics focus on protecting individuals from harm and injustice. Taken together, community and life form the dual core of Akan moral reasoning, with significant implications for applied ethical debates concerning issues such as bioethics, justice, and social responsibility (Metz, 2017).

In relation to the concept of cultural self-construal, as articulated by Markus and Kitayama (1991), individuals define themselves in relation to others, particularly along the contrast between independent and interdependent orientations. Although this framework has been most prominently discussed in East Asian contexts, it is conceivable that similar dynamics may also arise within other cultural traditions. One such example can be observed among the Akan people of Ghana, whose social identity, kinship structures, and communal norms play a fundamental role in shaping personal self-understanding. Given the centrality of lineage, extended family ties, and community obligations in Akan society, patterns resembling interdependent self-construal are likely to emerge, though these are embedded within a distinctive historical and socio-cultural context that could explain differences in these societies.

### A discussion of psychometric quality of MFQ

Measurements from the instrument of the MFQ are debated. A review by Zakharin and Bates (2021) evaluating the psychometric quality of the MFQ-1 on recent results at that time concludes that the first postulated five theoretical dimensions can be best mapped in the data. However, there exist few studies that prefer models with the higher-order factors of individualizing and binding moral value orientation, such as Smith et al. (2017), as well as Vainio and Mäkiniemi (2016), which use a hierarchical structure, such as Wormley et al. (2023). Atari et al. (2023) suggest the MFQ-2 with an adjusted structure using a six-factor model with a better model fit (*CFI* = 0.979, *TLI* = 0.978, *RMSEA* = 0.024, *SRMR* = 0.023). The main adjustments from MFQ-1 to MFQ-2 are that the fairness dimension was split into equality and proportionality, and that the item formats were changed: there is no longer a distinction between the items “judgment” and “relevance” as in the MFQ-1. Instead, a single prompt “please indicate how well each statement describes you or your opinions” is now used for all items (Zakharin and Bates, 2023).

Researchers elaborate arguments that limit the adequate measurement results of the MFQ in the postulated factor solution: Zakharin and Bates (2021) state that a satisfactory model fit is not achieved. A further criticism is that these models fail to reflect the individualizing and binding moral value orientations, which are part of moral foundations theory. Another argument is that the number of dimensions cannot be mapped consistently, such as sub-components (Zakharin and Bates, 2023) or alternative factor solutions (Curry et al., 2019; Harper and Rhodes, 2021). Finally, there exist general influencing factors on responding behavior that follow a general morality factor or response bias, such as social desirability.

Currently, the MFQ-2 instrument is being analyzed in different ways. Results by Zakharin and Bates (2023) support the six-dimensional structure with the split of loyalty into separate factors for a group and a nation component for a sample in the U.S. and the UK. The analysis by Dogruyol et al. (2024) supports the six-dimensional structure in the Turkish context.

Additionally, researchers are exploring differences in the MFQ for gender that could be explained by social role theory (Eagly and Wood, 2012). Redlawsk et al. (2024) currently conclude for the general population that women score higher on the foundations of care and fairness. Skalski-Bednarz et al. (2023) corroborate these results for young adult Catholics in Poland, aged 19–25, while men score higher on the foundations of loyalty and authority. Shirai and Watanabe (2024) and Niazi et al. (2020) present results showing higher scores for females in the foundations of fairness and care. Further research by Röhm et al. (2022) discusses the meaning of sex in morality and shows that the stigmatization of vulnerable persons in drug use is largely influenced by the type of substance addiction and respondents’ moral value orientation.

To date, researchers postulate the analysis of measurement invariance for the MFQ, considering, for example, demographic variables (Nilsson, 2023). This is seen as necessary because comparing mean scores is often done without reflection on measurement quality between subgroups. For example, results on measurement invariance by gender reveal inconsistent results (Davies et al., 2014; Nilsson, 2023).

### Hints on criterion validity of MFQ

The instruments MFQ-1 and MFQ-2 are associated with people’s behavior. Consequently, there exist correlations for the use of external criteria based on research findings. Here, we limit our focus to the three components of (1) religiosity, (2) political attitude, and (3) pro-environmental actions.

Empirical results show an association between religiosity and MFT. Following Reynolds et al. (2020) and Yi and Tsang (2020), the binding moral value orientations are correlated with religiosity. These findings are similar across different religious groups: For example, Mikani and Rasoolzadeh Tabatabaei (2021) show associations between the binding moral value orientations and religious fundamentalism among Muslims in Iran. Greenway et al. (2019) have similar findings for a sample of Christians in the U.S.

Researchers have identified associations between MFT and political attitudes. Graham et al. (2009) suggest that the moral foundations of harm/care and fairness/reciprocity, which constitute peoples’ individualizing moral value orientation, correlate with liberal orientation, while the other three foundations (ingroup/loyalty, authority/respect, and purity/sanctity) correlate with conservative attitudes. A meta-analysis by Kivikangas et al. (2021) shows similar results: the foundations of care and fairness are negatively correlated with political conservatism, whereas authority, loyalty, and purity are positively correlated. A study from New Zealand shows that authority and purity correlate positively with political conservatism (Davies et al., 2014). Another study from Finland indicates that the individualizing moral value orientation—foundations of care and fairness—is negatively associated with right-wing orientation, while the binding moral value orientation—dimensions of loyalty, authority, and purity—is positively associated with right-wing orientation (Vainio and Mäkiniemi, 2016).

The moral foundations of care and fairness are predictors of pro-environmental actions. Welsch (2020) demonstrates that care and fairness foundations are linked to many different climate-friendly behaviors, based on data from the European Social Survey (ESS). Dickinson et al. (2016) find similar results using data from the Cornell National Social Survey in the U.S. The study by Skalski-Bednarz et al. (2023) provides deeper insights with a sample of 616 young adult Catholics from Poland, aged 19–25. They show that the care and fairness foundations are positively associated with environmental concern and ecological behavior. Further research from New Zealand (Milfont et al., 2019) and Finland (Vainio and Mäkiniemi, 2016) supports these findings.

## The present research

We aim to examine whether the MFQ-2 provides a valid and reliable measurement of moral foundations within the Ghanaian population, as an example of a non-WEIRD country. The aims of this paper are for the MFQ-2 (1) to evaluate the construct validity for a hierarchical model, (2) to test construct validity with gender as well as criterial validity with religiosity, and (3) to test the fairness of the MFQ-2 regarding gender, to ensure that the test functions equivalently across different groups. The prospective teachers in Ghana can be considered a small yet representative sample of the population.

The latest version of the MFQ-2, developed by Atari et al. (2023), is designed as a tool to measure moral foundations from a universalistic perspective. Currently, less research has investigated the psychometric quality of this new measurement instrument, with a few exceptions (Dogruyol et al., 2024; Zakharin and Bates, 2023). Consequently, researchers are faced with many challenges. A discussion is pending on whether the MFQ-2 is adequate for use in non-WEIRD countries (Dogruyol et al., 2024). Whereas analyses of the six moral foundations in the MFQ-2 have been conducted, the hierarchical factor structure has been less scrutinized, and there is a need for more investigation into the existence of the individualizing and binding moral value orientations for their importance in MFT. A further challenge arises in the analysis of measuring invariance in subgroups (Nilsson, 2023), since subgroups are often compared without reflection on test fairness, e.g., in demographics. Researchers have also shown that there are associations between moral foundations and gender (Redlawsk et al., 2024), as well as between moral foundations and religiosity (Reynolds et al., 2020; Yi and Tsang, 2020).

Due to the lack of comprehensive, population-based data in Ghana, the present study draws on pre-service teachers as a proxy group. Although this approach does not yield fully representative insights into the broader population, it nonetheless offers a valuable empirical basis. Within the constraints of the available data infrastructure, this strategy enables us to generate evidence that would otherwise remain inaccessible and thereby advances an underexplored area of psychological research. In line with Neugebauer (2019), it is argued that teachers are responsible for transmitting knowledge, skills, and values to students or the next generation of a country. The teachers’ assessment of students’ performance by grades, certificates, and track recommendations regulates access to the next levels of the education system as well as occupational positions. Consequently, teachers have a selective function for society, and their communicated norms are seen as important for a nation. Pre-service teachers in Ghana are well-suited for a psychometric examination of a moral measurement instrument because they possess advanced education, are familiar with educational and cultural contexts, and represent a focused, relevant subgroup where theoretical constructs can be meaningfully assessed without requiring the entire population (Nyamekye et al., 2024).

Following the Varkey Foundation (2018), Ghana ranks 32nd out of the 35 countries surveyed with respect to teacher status, positioning Ghanaian teachers among those with the lowest levels of societal esteem internationally. This ranking underlines the comparatively limited prestige associated with the profession in Ghanaian society. At the same time, the survey reveals a remarkable discrepancy between external perceptions of the profession and school-level dynamics: while general respect for teachers is relatively low at the societal level, nearly three-quarters of Ghanaians (70%) nonetheless believe that pupils respect their teachers, which represents the fourth-highest rate across all countries included in the study. This tension—between limited societal recognition of the profession on the one hand and comparatively high perceptions of student respect on the other—offers a nuanced picture of teachers’ social position in Ghana and, crucially, demonstrates that their role must be understood within the specific cultural and educational context rather than through global generalizations.

## Methods

### Participants and design

We re-analyzed data from 1,049 pre-service teachers, students who are currently enrolled in a teacher education program working toward becoming certified teachers but have not yet begun full professional teaching, at the University of Education Winneba in Ghana (see Winkelkotte et al., 2025, for the original data set). Winkelkotte et al. (2025) conducted a pen-and-paper survey from May to July 2022. Researchers used a cross-sectional design with convenience sampling. The project followed privacy policy, and all participants were informed about their voluntary attendance without any incentives. The research was embedded in a larger study program that elaborates on the research gap regarding the attitudes of pre-service teachers towards inclusion and the relations between morality and stigmatization.

The students in the sample were enrolled in a study program for bachelor’s and master’s degrees for prospective teachers, with an average age of *M* = 24.13 years (*SD* = 4.17). The sample includes 55 percent male and 45 percent female students. The proportion of ethnic groups in the sample was half Akan (50 percent), about 13 percent Ewe, nearly 6 percent Ga-Dangme, and the remaining students belonged to groups that had a proportion below 5 percent of their group. Inspecting religious group affiliation, about 79 percent were Christians, approximately 8 percent were Muslims, and the remaining groups were each represented by less than 5 percent.

### Measures

Participants completed the MFQ-2 with the original 36 items in the English language, which is also an official language in Ghana, with six items for every moral foundation. A five-point Likert scale was used, varying from 1 (= does not describe me at all) to 5 (= describes me extremely well), with the following introduction: “For each of the statements below, please indicate how well each statement describes you or your opinions.” (Supplementary Table 1 describes the item text). In Table 1, the first descriptive statistics and the reliability of the MFQ-2 scales are shown. The reliability for the individualizing moral value orientation (*α* = 0.40; *ω* = 0.45) and for the purity foundation (*α* = 0.58; *ω* = 0.55) is low, but it can be considered high for the binding moral value orientation (*α* = 0.85; *ω* = 0.86; Viladrich et al., 2017, see reliability >0.70 as acceptable). The binding moral value orientation (*kurtosis* = 2.75), the loyalty foundation (*kurtosis* = 2.66), and the authority foundation (*kurtosis* = 3.30) revealed problematic kurtosis that indicates difficulties with normal distribution (Hair et al., 2014).

**Table 1:** Descriptive statistics and reliability of MFQ-2 scales.

	Descriptive statistics and reliability						
	Number of items	*M*	*SD*	*α*	*ω*	Skewness	Kurtosis
Individualizing value orientation	2^a^	3.74	0.57	0.40	0.45	−0.42	0.39
Care	6	4.20	0.65	0.74	0.74	−1.07	1.44
Equality	6	3.28	0.79	0.67	0.68	−0.29	−0.11
Binding value orientation	4^b^	4.14	0.54	0.85	0.86	−1.30	2.75
Proportionality	6	4.03	0.68	0.66	0.65	−0.74	0.42
Loyalty	6	4.30	0.64	0.73	0.73	−1.43	2.66
Authority	6	4.31	0.61	0.71	0.73	−1.51	3.30
Purity	6	3.90	0.66	0.58	0.55	−0.72	0.53

Total sample size is *N* = 1,049. ^a^ Two foundations: care and equality. ^b^ Four foundations: proportionality, loyalty, authority, and purity.

Data on religiosity (*M* = 8.14; *SD* = 2.18) was collected using one single item. The item text is, “Please indicate how religious you would describe yourself.” Participants could choose on a 10-point Likert scale between 1 (= not religious at all) and 10 (= absolutely religious).

### Data analyses and missing values

**Figure 1:**
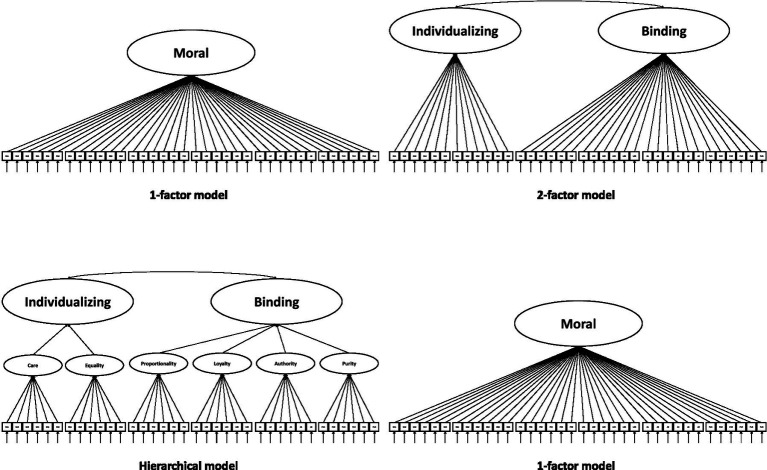
Estimated models for analyzing factor structure.

All analyses were conducted using the software R (R Core Team, 2021) version 4.3.2. In the first step, we analyzed the construct validity of the MFQ-2. For this reason, we estimated four different measurement models for evaluating model fit (see Figure 1 for details) using confirmatory factor analysis (CFA). The R syntax is provided in Supplementary material. The problems described with the normal distribution prompted us to use the weighted least squares mean and variance-adjusted (WLSMV) estimator (Li, 2016). We followed the criteria by Hu and Bentler (1999) that indicate *RMSEA* ≤ 0.06, *CFI* and *TLI* ≥ 0.95, as well as *SRMR* ≤ 0.08 as adequate model fit. We evaluated the models according to model fit as well as theoretical reasons. We used the package “lavaan” (Rosseel, 2012) version 0.6.18 for our analyses.

The criteria validity is evaluated through the MFQ-2 instrument and its association with religiosity as well as gender for construct validity. For a first inspection, correlations (*r*) according to Pearson estimated using values from 0.10 to 0.29 are considered small, between 0.30 and 0.49 as medium, and ≥0.50 as large (Cohen, 1988). A further analysis for gender is done by using *t*-tests for independent samples, with an effect size of *Cohen’s d* from 0.20 to 0.49 considered small, between 0.50 and 0.79 as medium, and ≥0.80 as large (Cohen, 1988). For these analyses, the package “psych” (Revelle, 2024) version 2.4.6.26 was used. Multiple regression is used for analyzing the MFQ-2 dimensions on the dependent variable religiosity. A k-fold cross-validation, in our analysis, where we use five folds, is further conducted for evaluating the results through *root mean squared error* (RMSE) and *mean absolute error* (MAE). Lower values for RMSE and MAE indicate a better fit as well as prediction accuracy (Hastie et al., 2009; Karunasingha, 2022; Kim and Kim, 2016; Olsen and Delen, 2008). The packages “caret” (Kuhn, 2008) version 6.0.94, “ModelMetrics” (Hamner et al., 2018) version 1.2.2.2, and “Metrics” version 0.1.4 were used. Significance is seen as *p* < 0.05 (two-tailed).

The evaluation of test fairness of the MFQ-2 regarding gender is done through testing measurement invariance. We are following the four steps from Putnick and Bornstein (2016, p. 73): “(1) configural, equivalence of model form; (2) metric (weak factorial), equivalence of factor loadings; (3) scalar (strong factorial), equivalence of item intercepts or thresholds; and finally (4) residual (strict or invariant uniqueness), equivalence of items’ residuals or unique variances.” We use the cut-off values suggested by Chen (2007) and interpret a decline of *CFI* ≤ −0.010 and *RMSEA* ≥ 0.015 as a hint for non-invariance. The package “semTools” (Jorgensen et al., 2022) version 0.5.6 was used.

The issue of determining the appropriate level of measurement invariance for cross-group comparisons remains highly contested. On the one hand, Nießen et al. (2020) insist on strict criteria, arguing that scalar invariance constitutes a necessary condition for the meaningful comparison of latent means, while strict invariance is indispensable for comparisons at the manifest level, whether item- or scale-based. This perspective reflects a strong commitment to methodological rigor, yet it also imposes demanding requirements that are difficult to meet in applied research. In contrast, other scholars have questioned whether such stringent standards are warranted for most psychological applications. For instance, Fischer and Poortinga (2018), Fischer and Karl (2019), Fischer et al. (2022), Fischer and Rudnev (2024) emphasize that scalar invariance may not be essential in many contexts, suggesting that configural or metric invariance is often sufficient to address substantive psychological questions. The tension between these positions reflects deeper disagreements about the balance between methodological idealism and empirical practicality: whereas the former prioritizes strict comparability as a precondition for inference, the latter highlights the risk of discarding meaningful findings when rigid criteria cannot be satisfied. Further complicating the debate, novel methodological developments have expanded the toolkit for testing measurement invariance beyond the traditional multi-group confirmatory factor analysis framework. The observation of systematic bias points to meaningful cultural variation that is not yet understood. Stricter (multi-group CFA) and more flexible approaches to testing for invariance (Fischer and Rudnev, 2024; Wurster, 2022), differential item functioning (DIF), and projection-based procedures are tools to understand this variation.

In the data, there are missing values. Analyses of item non-response in the variables range between 0.7 and 16.2% (*M* = 2.33; *SD* = 2.77). In the data, 38,982 measurements (97.66% of the sample) and 674 cases (64.25% of the sample) had no missing values. In further analyses, we estimate the missing data through the method of multiple imputation by chained equations using the R package “mice,” version 3.16.0, with 100 imputations (van Buuren and Groothuis-Oudshoorn, 2011).

## Results

### Construct validity of the factor structure

In a first step, we estimated the models shown in Figure 1 for analyzing construct validity. Table 2 shows the model fit, while Supplementary Table 3 includes the factor loadings with intercorrelations (see Supplementary Tables 4, 5). The interpretation of the results indicates that the models “Hierarchical model” (*χ^2^* = 1754.29, *df* = 587, *p* < 0.001; *CFI* = 0.978; *TLI* = 0.977; *RMSEA* = 0.044; *SRMR* = 0.053) and “6-factor model” (*χ^2^* = 1682.82, *df* = 579, *p* < 0.001; *CFI* = 0.980; *TLI* = 0.978; *RMSEA* = 0.043; *SRMR* = 0.052) fit similarly and show the best model fit. Results indicate that the first-order factor structure demonstrates an adequate fit, whereas no clear improvement emerges at the second-order level. However, there exists a strong intercorrelation between individualizing and binding moral value orientation with *r* = 0.97 (latent correlation; see Supplementary Table 4) and a manifest correlation of *r* = 0.64, which is problematic. Factor loadings of the second-order components are *λ* > 0.90, except for equality where *λ* = 0.41.

**Table 2:** Model fit comparisons for the estimated models.

	Statistics							
	*χ* ^2^	*df*	*p*	*χ*^2^/*df*	CFI	TLI	RMSEA	SRMR
Hierarchical model	1,754.29	587	<0.001	2.99	0.978	0.977	0.044	0.053
Six-factor model	1,682.82	579	<0.001	2.91	0.980	0.978	0.043	0.052
Two-factor model	2,862.07	593	<0.001	4.83	0.958	0.955	0.060	0.063
One-factor model	2,993.30	594	<0.001	5.04	0.956	0.953	0.062	0.064

*N = 1,049.*

### Criterion validity of religiosity and construct validity of gender

The analyses start by inspecting correlations (*r*) of MFQ-2 with gender and religiosity (see Table 3). The results show that all correlations for gender are negative, meaning that female students score higher, except for loyalty. The strongest correlations are estimated for gender with the individualizing moral value orientation (*r* = −0.09), the care foundation (*r* = −0.13), and the purity foundation (*r* = −0.08). Correlations between the MFQ-2 and religiosity are all positive. The strongest correlations are found for religiosity within the purity foundation (*r* = 0.19), the binding moral value orientation (*r* = 0.14), and the proportionality foundation (*r* = 0.10).

**Table 3:** Correlation (*r*) of MFQ-2 components with gender and religiosity.

	Correlations (r)	
	Gender	Religiosity
Individualizing value orientation	−0.09**	0.09**
Care	−0.13***	0.04
Equality	−0.03	0.09**
Binding value orientation	−0.03	0.14***
Proportionality	−0.04	0.10**
Loyalty	0.02	0.09*
Authority	−0.01	0.07*
Purity	−0.08*	0.19***

*N* = 1,049. Gender is seen as female (= 0) and male (= 1). *p < 0.05, **p < 0.01, ***p < 0.001.

Further analyses of the MFQ-2 for construct validity with gender using *t*-tests underline the analyses of the correlations (*r*). See details in Table 4. Female students always score higher, except for the dimension of loyalty. Significant small effects are estimated for the care dimension (*t*(1047) = 4.11, *p* < 0.001, *d* = 0.25). There are also significant effects for the individualizing moral foundation (*t*(1047) = 2.94, *p* = 0.003, *d* = 0.18) and the purity dimension (*t*(1047) = 2.51, *p* = 0.012, *d* = 0.16).

**Table 4:** Comparisons between gender and MFQ-2 components.

	Statistics							
	Female		Male		*df*	*t*	*p*	*d*
	M	SD	M	SD				
Individualizing value orientation	3.80	0.56	3.69	0.57	1,047	2.94	0.003	0.18
Care	4.29	0.57	4.12	0.70	1,047	4.11	<0.001	0.25
Equality	3.31	0.80	3.26	0.78	1,047	0.89	0.373	0.06
Binding value orientation	4.15	0.51	4.12	0.56	1,047	0.98	0.325	0.06
Proportionality	4.05	0.68	4.00	0.68	1,047	1.22	0.220	0.08
Loyalty	4.28	0.63	4.32	0.65	1,047	0.79	0.430	0.05
Authority	4.32	0.56	4.31	0.65	1,047	0.23	0.813	0.01
Purity	3.96	0.63	3.86	0.68	1,047	2.51	0.012	0.16

*N = 1,049. d = Cohen’s d.*

We further investigate the analyses of multiple regressions to elaborate on validity. In detail, we analyze associations between the MFQ-2 components and religiosity. The results mostly confirm the analyses that were already conducted in the correlation analysis in Table 3. Specifically, we estimated three models that are presented in Table 5. Model 1 integrates the foundations of care and equality. In Model 2, the other four components of the six-factor model were included to check the robustness of the effects from Model 1. Model 3 integrates the individualizing and binding moral value orientations. Model 1 shows a significant association from the equality foundation (*ꞵ* = 0.09; *p* = 0.007) on religiosity. Model 2 shows significant results for the purity foundation (*ꞵ* = 0.24; *p* < 0.001) as well as a surprisingly negative significant result for the care foundation (*ꞵ* = −0.12; *p* < 0.001). In Model 3, a significant result is estimated for the binding moral value orientation (*ꞵ* = 0.13; *p* < 0.001). Explained variance is in the models *adj. R^2^* ≤ 0.04. A small increase in explained variance is noted from Model 1 (*R^2^* = 0.01) to Model 2 (*R^2^* = 0.04) with *Δ adj. R^2^* = 0.03.

**Table 5:** Results on predicting religiosity using multiple regression and cross-validation.

	Statistics								
	Model 1			Model 2			Model 3		
	*ꞵ*	SE	*p*	*ꞵ*	SE	*p*	*ꞵ*	SE	*p*
Care	0.02	0.11	0.491	−0.12	0.15	0.008			
Equality	0.09	0.09	0.007	0.05	0.09	0.156			
Proportionality				0.04	0.13	0.304			
Loyalty				0.02	0.16	0.624			
Authority				−0.04	0.18	0.392			
Purity				0.24	0.13	<0.001			
Individualizing value orientation							0.00	0.15	0.936
Binding value orientation							0.13	0.16	<0.001
Intercept	7.05	0.47	<0.001	6.07	0.53	<0.001	5.87	0.54	<0.001
*Adj. R^2^*		0.01			0.04			0.02	
*RMSE*		*M* = 2.18 *SD* = 0.06		*M* = 2.16 *SD* = 0.09			*M* = 2.16 *SD* = 0.12		
*MAE*		*M* = 1.65 *SD* = 0.06		*M* = 1.64 *SD* = 0.09			*M* = 1.65 *SD* = 0.06		

*N = 1,049. Adj. R2 = adjusted R2. RMSE, root mean squared error; MAE, mean absolute error. ꞵ = standardized beta. Cross-validation is a k-fold cross-validation with five folders.*

The results of the k-fold cross-validation are shown in Table 5. The estimated coefficient of *RMSE* of *M* > 2 indicates a questionable quality of the models. The *MAE* for all models is between *M* = 1.64–1.65, which indicates a slightly better, but still moderate, informative quality of the models. The estimations of *RMSE* and *MAE* show consistent results (*SD* = 0.06–0.12).

Analyses provide initial evidence that the examination of criterion validity in relation to religiosity and construct validity in relation to gender in the MFQ-2 offers early indications of the measure’s validity. These findings suggest that certain patterns of association align with theoretical expectations. Overall, the results provide initial empirical evidence for a weakly supported general validity of the MFQ-2.

### Measurement invariance on gender

Measurement invariance is tested based on gender. Results are shown in Table 6. Criteria for strict invariance in comparing different models are hit (∆ *CFI* > −0.010 and ∆ *RMSEA* < 0.015). Specifically, there are values for ∆ *CFI* from −0.006 to −0.001 and ∆ *RMSEA* from −0.001 to 0.003. Consequently, strict invariance is achieved, and mean differences can be compared without restriction (Nießen et al., 2020).

**Table 6:** Results of measurement invariance testing between gender.

	Statistics								
	*χ* ^2^	*df*	CFI	RMSEA	∆*χ*^2^	∆*df*	∆CFI	∆RMSEA	*p*
Configural invariance	1,710.29	1,174	0.967	0.033					
Metric invariance	1,667.52	1,208	0.961	0.036	42.77	34	−0.006	0.003	0.011
Scalar invariance	1,703.35	1,236	0.960	0.036	35.83	28	−0.001	0.000	0.011
Strict invariance	1,737.60	1,272	0.959	0.035	34.25	36	−0.001	−0.001	0.118

*N = 1,049. Gender is seen as male and female.*

## Discussion

The purpose of our paper is to shed light on the measurement quality of the recently published MFQ-2 (Atari et al., 2023). The present re-analysis replicated and extended the current state of research. To date, there have been few analyses of the quality of this measurement instrument, such as Zakharin and Bates (2023), Dogruyol et al. (2024), or Frisari et al. (2025), indicating that further reviews are necessary. A key question is the analysis of the MFQ-2 in non-WEIRD countries (Henrich et al., 2010), such as Ghana. For theoretical reasons, it is essential to analyze the factor structure with a special focus on the individualizing and binding moral value orientations. In the MFQ-1, analyses of six-factor models are preferred over hierarchical models (Zakharin and Bates, 2021). Like its predecessor, the MFQ-1, the MFQ-2 must be tested for criterion validity and construct validity. Here, gender and religiosity are known to be correlating criteria (Skalski-Bednarz et al., 2023; Yi and Tsang, 2020). Consequently, efforts must also be made to examine how gender and religiosity are related to the components of the MFQ-2. Often, means in subgroups are compared, but it is not clarified if the measurement quality in these groups is the same.

The authors’ empirical analyses of the structure of the models show similar findings for the hierarchical model and the six-factor model regarding construct validity in this sample of pre-service teachers in Ghana. The current state of research about the MFQ-2 is not well elaborated and brings no consistent results because some researchers use hierarchical models (Zakharin and Bates, 2023), while others prefer six-factor models (Dogruyol et al., 2024). These empirical results can be interpreted in different ways. A hierarchical solution with broader dimensions such as “binding” and “individualization” does not provide a better model fit than the six-factor model of the MFQ-2, due to psychometric and data-related challenges. Both models may fail because the initial first-order factors are mis-specified, the models are too complex, or a small sample size is used (Fan and Sivo, 2005; Zheng and Bentler, 2024). Furthermore, the MFQ-2 was developed within Western contexts that may not fully capture the moral realities of Ghanaian culture, where other principles or moral foundations may be overlooked. Etic and emic challenges exist here; the etic perspective refers to an outsider’s objective view analyzing behaviors across cultures, while the emic perspective captures the insider’s subjective experience within a specific culture (Cheung et al., 2011). This study is conducted with an etic perspective, and so this circumstance of cultural divergence can distort factor structures, as items reflecting loyalty, authority, or care may be interpreted differently in Ghana, leading to a poor model fit regardless of complexity. Moreover, the hierarchical split between “binding” and “individualizing” foundations is viewed differently in Ghana, blurring distinctions. Language and interpretation issues further complicate matters, as subtle semantic differences can affect item comprehension. Variations in education and moral socialization could influence the results. Finally, teachers are often oriented in practical ways rather than abstract dimensions.

The estimation of low reliability, as observed for individualizing value orientation (*α* = 0.40) and purity (*α* = 0.58), poses a problem. It is possible that the underlying dimensions exist but are not adequately captured by the items employed, which may indicate an issue of measurement equivalence. Such discrepancies could be attributable to cultural influences or differences in language use. An examination based on Differential Item Functioning between WEIRD and non-WEIRD countries could provide valuable insights in this regard. Further analyses on criterion and construct validity are conducted against the background of the current state of research, which shows that the binding moral value orientation, along with its related foundations, is associated with religiosity (Reynolds et al., 2020; Yi and Tsang, 2020) and that females have a higher individualizing moral value orientation (Redlawsk et al., 2024; Skalski-Bednarz et al., 2023). The authors’ empirical results confirm these findings and additionally show that females score higher in the care foundation compared to males. The higher score in the purity foundation is not expected. The authors’ results for religiosity confirm previous findings and show an association with the purity foundation and the binding moral value orientation. This finding is also underpinned by the fact that a small increase in the explained variance is noted from Model 1 (*R^2^* = 0.01) to Model 2 (*R^2^* = 0.04) with *Δ adj. R^2^* = 0.03, indicating an “incremental validity” of the integrated variables of the binding moral value orientation. The authors’ cross-validation reveals limited predictive validity of the predictors for religiosity.

When the results in this study are considered from relativist and universalist perspectives in general (Berry et al., 2011; Poortinga, 2021), it becomes apparent that a relativist perspective risks overlooking universal psychological constructs underlying the MFQ-2 by emphasizing cultural specificity, which can lead to fragmented or inconsistent validity across contexts. Conversely, a universalist perspective may inadequately account for cultural nuances in Ghana, potentially resulting in the instrument being only moderately valid due to ignoring culturally specific interpretations of moral values and behaviors. An adequate solution and way forward could be to consider a combined emic/etic perspective to appropriately assess different perspectives (Cheung et al., 2011).

Inconsistent results exist in measurement invariance for gender. The authors’ research contributes to the evidence that measurement invariance for gender exists. Results from women and men are comparable based on measurement quality. We confirm the work of previous research, such as Nilsson (2023).

Our research elaborates on the validity of the MFQ-2 for a non-WEIRD country. There are initial indications that the MFQ-2 is an acceptable measurement instrument for Ghana. Our investigation also revealed many signs of fundamental problems in the MFQ-2 for the region of Ghana. The following examples are representative. The reliability coefficient for scales is often below 0.70 (e.g., individualizing moral value orientation and purity foundation), which is not in line with the demands of the current state of research quality (Viladrich et al., 2017). Researchers have stated that purity has a wide variety of interpretations (Gray et al., 2023) and also suggest the independence of this dimension (Kollareth and Russell, 2023). Gray et al. (2023) provided an overview of the meanings of purity, including historical and psychological contexts, and classified nine dimensions. The empirical differentiation between a six-factor model and a hierarchical model solution is not sufficient, but the factor structure seems to be adequate. The association with external criteria and construct validation of gender is limited because there are surprising results that do not align with previous findings, such as females scoring higher in the purity foundation compared to men.

Our findings must also be interpreted in the broader context of the concept of cultural self-construal as articulated by Markus and Kitayama (1991). This framework, which distinguishes between independent and interdependent models of the self, has become a central paradigm in understanding how cultural settings shape psychological orientations and moral reasoning. While most work has focused on comparisons between East Asian and Western cultural contexts, there are potential intersections worth exploring between East Asian and African societies. Preliminary anthropological and psychological evidence suggests that certain African cultural settings also emphasize relationality, communal integration, and moral obligations toward the group—features commonly associated with interdependent self-construals (Nsamenang, 2002; Heine, 2010). Systematic cross-cultural studies directly comparing East Asian and African contexts remain scarce. Such comparative research would be highly desirable in order to evaluate how cultural self-construal manifests within the framework of moral foundations theory (Haidt and Joseph, 2004). Specifically, it could clarify how dimensions such as individualizing foundations (e.g., care and fairness) and binding foundations (e.g., loyalty, authority, and sanctity) are prioritized or negotiated across these cultural landscapes. This would not only allow for a deeper understanding of similarities and differences but also contribute to theorizing higher-order cultural patterns of individualization and binding, and how these are embedded within distinct cultural ecologies.

From a practical perspective, our findings suggest that the extent to which it is useful to differentiate between the six underlying moral foundations or, alternatively, to focus on the higher-order distinction between individualizing and binding moral orientations depends on the specific situational and cultural context in which social behavior and underlying motives are to be understood.

For example, the understanding of purity may differ depending on the cultural context, which necessitates a specific consideration of this individual moral foundation in different cultural contexts (e.g., Gray et al., 2023). Depending on the situation, more variance in moral decisions may be explained by the characteristics of the six moral foundations (e.g., political attitudes and the purity foundation: Leota et al., 2023). For more general classifications and the understanding of larger (social) phenomena such as ideological polarization (e.g., Malka et al., 2016), counseling candidates (e.g., Kılıç, 2024), or climate-friendly choices (Vainio and Mäkiniemi, 2016), it seems to be appropriate to first understand the coarser motives (i.e., whether the reasoning/behavior is based more on a focus on the individual or the group). In general, more research is needed on the connections between social behavior, social phenomena, and moral foundations based on MFT, especially against the background of influencing factors such as gender, religiosity, and different cultural contexts.

Our research has some limitations. There are strong intercorrelations between the second-order factors of individualizing and binding moral value orientations that indicate problems for unbiased measurements. We measure religiosity with only one item, which is a problem for measurement quality. The use of a validated test with multiple items for better measurement quality is desirable. The representativeness of the sample, as we only collected data from pre-service teachers, is also debatable since it is a specific subgroup.

As highlighted in the Measures section, morality in our study is assessed through self-reports. As noted by Demetriou et al. (2015), the use of self-reports entails several methodological limitations. First, responses may be invalid due to participants providing inaccurate or socially desirable answers, particularly in the case of sensitive topics (social desirability bias). Second, response bias may occur when participants respond in a patterned manner, such as endorsing all items positively or negatively, regardless of item content. A further challenge relates to the wording and clarity of questionnaire items, which may invite divergent interpretations. In addition, simply taking part in a research project can influence participants’ behavior and responses, a phenomenon referred to as the Hawthorne effect. Finally, the fixed and standardized scoring format restricts participants’ opportunity to convey the full range of their experiences and emotions (Wild et al., 2024). If one relates this to cultural differences, then the following can be concluded: cultural identity shapes how individuals perceive themselves and evaluate their own behavior, as it is formed through values, beliefs, traditions, and social practices conveyed within one’s culture of origin. These cultural frameworks influence not only self-perception but also the ways in which individuals present and assess themselves. Consequently, the interpretation of self-reports must acknowledge cultural variability, since the meaning and validity of responses are closely interlinked with cultural identity and its normative expectations.

The generalizability of the present findings is necessarily limited, as the sample represents a comparatively highly educated subgroup within the Ghanaian population. Consequently, the extent to which results extend to less educated or more rural populations remains uncertain and warrants future empirical examination across broader demographic strata.

Another potential limitation of the present study concerns the cultural transferability of the survey items employed. The questionnaire was originally developed and validated within an English-speaking, WEIRD (Western, Educated, Industrialized, Rich, and Democratic) cultural context. It is therefore possible that certain items do not carry the same connotations or pragmatic meanings when administered in Ghana, a non-WEIRD setting with distinct socio-cultural norms and communicative practices. Subtle differences in language use, value orientations, and culturally embedded constructs may have shaped how participants interpreted the items, potentially affecting the reliability and validity of their responses (International Test Commission, 2013). A semantic equivalence across cultural contexts is not guaranteed. Future research should invest in systematic cross-cultural validation procedures, such as cognitive interviewing and measurement invariance testing, to better assess whether the constructs are interpreted consistently across diverse populations and to strengthen the generalizability of findings.

One important limitation of this study lies in the reliance on an etic, or outside, perspective in the assessment of moral foundations in Ghana. While this approach allows for a comparative framework across cultural contexts, it inevitably risks obscuring locally specific meanings and practices. The application of the etic perspective was operationalized through the use of theoretical constructs and measurement tools developed predominantly within Western academic discourse, which were then applied to the Ghanaian context. This methodological choice may have shaped both the interpretation of participants’ responses and the overall conclusions drawn, as it prioritizes the analytical categories of the researcher over insider (emic) understandings. Consequently, the findings may not fully capture the nuances of moral reasoning as they are lived and articulated within Ghanaian cultural settings. This limitation highlights the need for further research that integrates emic perspectives or employs mixed approaches to more effectively bridge culturally specific insights with cross-cultural comparisons.

There is a methodological challenge in interpreting cultural differences that must be viewed through the lens of generalized response styles. These challenges manifest as systematic answer tendencies across groups, including socially desirable responding, extreme responding, and midpoint responding. Such variations occur systematically across cultural contexts and significantly influence survey outcomes (He and Van De Vijver, 2015).

Based on our research, new research questions arise. Consequently, it must be evaluated whether further moral foundations are necessary for integration into the MFQ-2. Another challenge is that the MFQ-2 must be analyzed more deeply for criterion validity, such as political orientation (Kivikangas et al., 2021; Milesi, 2017), attitudes toward migration (Captari et al., 2019), or pro-environmental behavior (Vainio and Mäkiniemi, 2016). In line with Nilsson (2023), it is postulated that analyses through measurement invariance must be conducted to ensure test fairness and to confirm that comparing means is appropriate for demographic variables as well as for sophisticated and vulnerable groups. Another promising avenue for future research lies in examining the reasons behind variations in moral foundations. In particular, it would be valuable to explore how the assessment of economic challenges, cultural factors, and institutional weaknesses may shape these differences. Furthermore, generational contrasts and socioeconomic conditions could play a crucial role in explaining why certain moral perspectives are more salient in some contexts than in others.

## Conclusion

The present work shows that the MFQ-2 in Ghana has moderate validity. The factor structure for a hierarchical model, as well as a 6-factor model, which are the best fitting models through different CFAs, is weakly supported. Initial indications suggest correlations with external criteria. Measurement invariance is established for gender. Further analyses are needed to evaluate the instrument against the background of MFT in non-WEIRD countries to overcome the limitations of this study.

## Data Availability

The raw data supporting the conclusions of this article will be made available by the authors without undue reservation.
