# Supplementary material for: Inspecting the factors of individualizing and binding moral value orientation in the Moral Foundations Questionnaire-2 for validation—a re-analysis of data from pre-service teachers in Ghana
Source: Front Psychol. 2025 Nov 26;16:1665536. doi: 10.3389/fpsyg.2025.1665536 (PMC12689288; doi:10.3389/fpsyg.2025.1665536)
Supplement: Supplementary file 1 [file Data_Sheet_1.docx]

Supplement Material

**Table 1**

*Itemtext of the measurement instrument MFQ-2*

| Item ID | Itemtext |  |
| --- | --- | --- |
|  | *Care* |  |
| CA1 | Caring for people who have suffered is an important virtue. |  |
| CA2 | I believe that compassion for those who are suffering is one of the most crucial virtues. |  |
| CA3 | We should all care for people who are in emotional pain. |  |
| CA4 | I am empathetic toward those people who have suffered in their lives. |  |
| CA5 | Everyone should try to comfort people who are going through something hard. |  |
| CA6 | It pains me when I see someone ignoring the needs of another human being. |  |
|  | *Equality* |  |
| EQ1 | The world would be a better place if everyone made the same amount of money. |  |
| EQ2 | Our society would have fewer problems if people had the same income. |  |
| EQ3 | I believe that everyone should be given the same quantity of resources in life. |  |
| EQ4 | I believe it would be ideal if everyone in society wound up with roughly the same amount of money. |  |
| EQ5 | When people work together toward a common goal, they should share the rewards equally, even if some worked harder on it. |  |
| EQ6 | I get upset when some people have a lot more money than others in my country. |  |
|  | *Proportionality* |  |
| PR1 | I think people who are more hard-working should end up with more money. |  |
| PR2 | I think people should be rewarded in proportion to what they contribute. |  |
| PR3 | The effort a worker puts into a job ought to be reflected in the size of a raise they receive. |  |
| PR4 | It makes me happy when people are recognized on their merits. |  |
| PR5 | In a fair society, those who work hard should live with higher standards of living. |  |
| PR6 | I feel good when I see cheaters get caught and punished. |  |
|  | *Loyalty* |  |
| LO1 | I think children should be taught to be loyal to their country. |  |
| LO2 | It upsets me when people have no loyalty to their country. |  |
| LO3 | Everyone should love their own community. |  |
| LO4 | Everyone should defend their country, if called upon. |  |
| LO5 | Everyone should feel proud when a person in their community wins in an international competition. |  |
| LO6 | I believe the strength of a sports team comes from the loyalty of its members to each other. |  |
|  | *Authority* |  |
| AU1 | I think it is important for societies to cherish their traditional values. |  |
| AU2 | I feel that most traditions serve a valuable function in keeping society orderly. |  |
| AU3 | I think obedience to parents is an important virtue. |  |
| AU4 | We all need to learn from our elders. |  |
| AU5 | I believe that one of the most important values to teach children is to have respect for authority. |  |
| AU6 | I think having a strong leader is good for society. |  |
|  | *Purity* |  |
| PU1 | I think the human body should be treated like a temple, housing something sacred within. |  |
| PU2 | I believe chastity is an important virtue. |  |
| PU3 | It upsets me when people use foul language like it is nothing. |  |
| PU4 | If I found out that an acquaintance had an unusual but harmless sexual fetish, I would feel uneasy about them. |  |
| PU5 | People should try to use natural medicines rather than chemically identical human-made ones. |  |
| PU6 | I admire people who keep their virginity until marriage. |  |

*Note.* A 5-point Likert scale from 1 (= does not describe me at all) to 5 (= describes me extremely well) with introduction “For each of the statements below, please indicate how well each statement describes you or your opinions.” Items are taken from Atari et al. (2023).

**Table 2**

*Descriptive statistics and reliability of MFQ-2 scales*

|  |  | Statistics | | | | | | |
| --- | --- | --- | --- | --- | --- | --- | --- | --- |
|  | Number of items | *M* | *SD* | α | ω | *Skewness* | *Kurtosis* |  |
| Individualising Value Orientation | 2^a^ | 3.74 | 0.57 | .40 | .45 | –0.42 | 0.39 |  |
| Care | 6 | 4.20 | 0.65 | .74 | .74 | –1.07 | 1.44 |  |
| Equality | 6 | 3.28 | 0.79 | .67 | .68 | –0.29 | –0.11 |  |
| Binding Value Orientation | 4^b^ | 4.14 | 0.54 | .85 | .86 | –1.30 | 2.75 |  |
| Proportionality | 6 | 4.03 | 0.68 | .66 | .65 | –0.74 | 0.42 |  |
| Loyalty | 6 | 4.30 | 0.64 | .73 | .73 | –1.43 | 2.66 |  |
| Authority | 6 | 4.31 | 0.61 | .71 | .73 | –1.51 | 3.30 |  |
| Purity | 6 | 3.90 | 0.66 | .58 | .55 | –0.72 | 0.53 |  |

*Note.* Total sample size is *N* = 1,049. ^a^ two foundations: care and equality. ^b^ four foundations: proportionality, loyalty, authority and purity.

**Table 3**

*Factor loadings (λ) of confirmatory factor analyses based from models in Figure 1*

|  | Factor loadings (λ) | | | | |
| --- | --- | --- | --- | --- | --- |
|  | 1-factor model | 2-factor model | 6-factor model | Hierarchical model |  |
| CA1 | .49 | .52 | .52 | .52 |  |
| CA2 | .54 | .58 | .58 | .58 |  |
| CA3 | .70 | .75 | .74 | .74 |  |
| CA4 | .60 | .64 | .64 | .64 |  |
| CA5 | .69 | .74 | .74 | .74 |  |
| CA6 | .65 | .70 | .69 | .69 |  |
| EQ1 | .24 | .26 | .56 | .56 |  |
| EQ2 | .27 | .30 | .61 | .61 |  |
| EQ3 | .30 | .33 | .57 | .57 |  |
| EQ4 | .32 | .35 | .67 | .66 |  |
| EQ5 | .31 | .33 | .50 | .51 |  |
| EQ6 | .11 | .13 | .34 | .34 |  |
| PR1 | .44 | .45 | .48 | .48 |  |
| PR2 | .47 | .47 | .51 | .51 |  |
| PR3 | .57 | .58 | .62 | .62 |  |
| PR4 | .62 | .63 | .68 | .68 |  |
| PR5 | .50 | .51 | .55 | .55 |  |
| PR6 | .49 | .50 | .53 | .53 |  |
| LO1 | .69 | .69 | .70 | .71 |  |
| LO2 | .54 | .54 | .55 | .55 |  |
| LO3 | .61 | .62 | .63 | .63 |  |
| LO4 | .62 | .63 | .64 | .64 |  |
| LO5 | .68 | .69 | .70 | .70 |  |
| LO6 | .65 | .66 | .67 | .67 |  |
| A1 | .60 | .60 | .61 | .61 |  |
| A2 | .52 | .52 | .53 | .52 |  |
| A3 | .76 | .77 | .78 | .78 |  |
| A4 | .49 | .49 | .49 | .49 |  |
| A5 | .71 | .72 | .72 | .72 |  |
| A6 | .73 | .73 | .74 | .74 |  |
| PU1 | .55 | .55 | .58 | .58 |  |
| PU2 | .68 | .69 | .73 | .73 |  |
| PU3 | .42 | .42 | .45 | .45 |  |
| PU4 | .34 | .34 | .36 | .36 |  |
| PU5 | .35 | .35 | .37 | .37 |  |
| PU6 | .46 | .47 | .49 | .49 |  |
| Care # |  |  |  | .93 |  |
| Equality # |  |  |  | .41 |  |
| Proportionality ## |  |  |  | .90 |  |
| Loyalty ## |  |  |  | .98 |  |
| Authority ## |  |  |  | .99 |  |
| Purity ## |  |  |  | .93 |  |

*Note.* CA = care; EQ = equality; PR = proportionality; LO = loyalty; A = authority; PU = purity. # = components for second order analysis of individualising; ## = components for second order analysis of binding; *N* = 1,049.

**Table 4**

*Intercorrelation of confirmatory factor analyses from Table 2 for 2-factor model and hierarchical model*

|  | Individualising Value Orientation | |
| --- | --- | --- |
|  | 2-factor model | Hierarchical model |
| Binding Value Orientation | .88 | .97 |

*Note.* *N* = 1,049.

**Table 5**

*Intercorrelation of confirmatory factor analyses from Table 2 for 6-factor model and manifest correlations*

|  | Intercorrelations (r) | | | | | |  |
| --- | --- | --- | --- | --- | --- | --- | --- |
|  | 1. | 2. | 3. | 4. | 5. | 6. |  |
| 1. Care | – | .25 | .57 | .65 | .68 | .59 |  |
| 2. Equality | .38 | – | .30 | .21 | .24 | .28 |  |
| 3. Proportionality | .83 | .44 | – | .60 | .59 | .50 |  |
| 4. Loyalty | .88 | .33 | .90 | – | .73 | .57 |  |
| 5. Authority | .90 | .34 | .88 | .99 | – | .57 |  |
| 6. Purity | .89 | .42 | .81 | .89 | .92 | – |  |

*Note.* *N* = 1,049. Above angle bisector line manifest correlations are shown and below latent correlations.

**Table 6**

*Results of measurement invariance testing between gender*

|  | Statistics | | | | | | | | |
| --- | --- | --- | --- | --- | --- | --- | --- | --- | --- |
|  | χ^2^ | *df* | *CFI* | *RMSEA* | ∆χ^2^ | ∆*df* | ∆*CFI* | ∆*RMSEA* | *p* |
| Configural invariance | 1710.29 | 1174 | .967 | .033 |  |  |  |  |  |
| Metric invariance | 1667.52 | 1208 | .961 | .036 | 42.77 | 34 | –0.006 | 0.003 | .011 |
| Scalar invariance | 1703.35 | 1236 | .960 | .036 | 35,83 | 28 | –0.001 | 0.000 | .011 |
| Strict invariance | 1737.60 | 1272 | .959 | .035 | 34.25 | 36 | –0.001 | –0.001 | .118 |

*Note.* *N* = 1,049. Gender is seen as male and female.

R-Syntax

# Load packages

#install.packages("lavaan", dependencies = TRUE)

library(lavaan)

library (semTools)

# Import data

datas = read.csv2("E:/…/xxx.dat", header = TRUE, sep="\t", dec=".", na.strings = "-99" )

head (datas)

summary(datas)

# Reliability

datas$Care <- rowMeans(datas[,c(2:7)], na.rm = T)

datas$Eqauality <- rowMeans(datas[,c(8:13)], na.rm = T)

datas$Proportionality <- rowMeans(datas[,c(14:19)], na.rm = T)

datas$Loyalty <- rowMeans(datas[,c(20:25)], na.rm = T)

datas$Authority <- rowMeans(datas[,c(26:31)], na.rm = T)

datas$Purity <- rowMeans(datas[,c(32:37)], na.rm = T)

modelcfa1 <- '

Individualizing_CFA =~ Care + Eqauality

Binding_CFA =~ Proportionality+Loyalty+Authority +Purity '

fit1 <- sem(modelcfa1 , data=datas, estimator = "WLSMV", ordered = T)

summary(fit1, standardized=TRUE, fit.measures=TRUE)

reliability(fit1)

modelcfa2 <- '

Care_CFA =~ CA_1 +CA_2+CA_3+CA_4+CA_5+CA_6

Eqauality_CFA=~ EQ_1+EQ_2+EQ_3+EQ_4+EQ_5+EQ_6

Proportionality_CFA =~ PR_1+PR_2+PR_3+PR_4+PR_5+PR_6

Loyalty_CFA=~ LO_1+LO_2+LO_3+LO_4+LO_5+LO_6

Authority_CFA =~ AU_1+AU_2+AU_3+AU_4+AU_5+AU_6

Purity_CFA =~ PU_1+PU_2+PU_3+PU_4+PU_5+PU_6 '

fit2 <- sem(modelcfa2 , data=datas, estimator = "WLSMV", ordered = T)

summary(fit2, standardized=TRUE, fit.measures=TRUE)

reliability(fit2)

datas$Individualizing <- rowMeans(datas[,c(41:42)], na.rm = T)

datas$Binding <- rowMeans(datas[,c(43:46)], na.rm = T)

library(psych)

describe(datas )

# Validity

# CFA / 1-Factor-Model

GFaktor <- 'General =~ CA_1 + CA_2 + CA_3 + CA_4 + CA_5 + CA_6 +

EQ_1 + EQ_2 + EQ_3 + EQ_4 + EQ_5 + EQ_6 +

PR_1 + PR_2 + PR_3 + PR_4 + PR_5 + PR_6 +

LO_1 + LO_2 + LO_3 + LO_4 + LO_5 + LO_6 +

AU_1 + AU_2 + AU_3 + AU_4 + AU_5 + AU_6 +

PU_1 + PU_2 + PU_3 + PU_4 + PU_5 + PU_6'

GFaktor<- sem(GFaktor, data=datas,estimator = "WLSMV", ordered = T)

summary(GFaktor, standardized=TRUE, fit.measures=TRUE)

# CFA / 6-factor-model

Model_CFA1 <- 'Care_CFA =~ CA_1 + CA_2 + CA_3 + CA_4 + CA_5 + CA_6

Equality_CFA =~ EQ_1 + EQ_2 + EQ_3 + EQ_4 + EQ_5 + EQ_6

Proportionality_CFA =~PR_1 + PR_2 + PR_3 + PR_4 + PR_5 + PR_6

Loyalty_CFA =~ LO_1 + LO_2 + LO_3 + LO_4 + LO_5 + LO_6

Authority_CFA =~ AU_1 + AU_2 + AU_3 + AU_4 + AU_5 + AU_6

Purity_CFA =~ PU_1 + PU_2 + PU_3 + PU_4 + PU_5 + PU_6'

CFA1 <- sem(Model_CFA1, data=datas,estimator = "WLSMV", ordered = T)

summary(CFA1 , standardized=TRUE, fit.measures=TRUE)

reliability(CFA1 )

# CFA / 2-factor-model

TwoFaktor_CFA <- 'Individual_CFA =~ CA_1 + CA_2 + CA_3 + CA_4 + CA_5 + CA_6 + EQ_1 + EQ_2 + EQ_3 + EQ_4 + EQ_5 + EQ_6

Binding_CFA =~ PR_1 + PR_2 + PR_3 + PR_4 + PR_5 + PR_6 +

LO_1 + LO_2 + LO_3 + LO_4 + LO_5 + LO_6 +

AU_1 + AU_2 + AU_3 + AU_4 + AU_5 + AU_6 +

PU_1 + PU_2 + PU_3 + PU_4 + PU_5 + PU_6'

TwoFaktor <- sem(TwoFaktor_CFA, data=datas,estimator = "WLSMV", ordered = T)

summary(TwoFaktor, standardized=TRUE, fit.measures=TRUE)

reliability(TwoFaktor)

# CFA / Second-Order-Classic

Hiererchisch_Classic <- 'Care_CFA =~ CA_1 + CA_2 + CA_3 + CA_4 + CA_5 + CA_6

Equality_CFA =~ EQ_1 + EQ_2 + EQ_3 + EQ_4 + EQ_5 + EQ_6

Proportionality_CFA =~PR_1 + PR_2 + PR_3 + PR_4 + PR_5 + PR_6

Loyalty_CFA =~ LO_1 + LO_2 + LO_3 + LO_4 + LO_5 + LO_6

Authority_CFA =~ AU_1 + AU_2 + AU_3 + AU_4 + AU_5 + AU_6

Purity_CFA =~ PU_1 + PU_2 + PU_3 + PU_4 + PU_5 + PU_6

Individual_CFA =~ Care_CFA + Equality_CFA

Binding_CFA =~ Proportionality_CFA + Loyalty_CFA + Authority_CFA + Purity_CFA'

Classic<- sem(Hiererchisch_Classic, data=datas,estimator = "WLSMV", ordered = T)

summary(Classic, standardized=TRUE, fit.measures=TRUE)

fitMeasures(Classic)

fitMeasures(CFA1)

anova(Classic, CFA1, method = "standard")

# Measurement invariance

fit.config <- cfa(Hiererchisch_Classic, data = datas, group = "Gender_r", estimator = "WLSMV")

fit.metric <- cfa(Hiererchisch_Classic, data = datas, group = "Gender_r", group.equal = "loadings", estimator = "WLSMV")

fit.scalar <- cfa(Hiererchisch_Classic, data = datas, group = "Gender_r", group.equal = c("loadings","intercepts"), estimator = "WLSMV")

fit.strict <- cfa(Hiererchisch_Classic, data = datas, group = "Gender_r", group.equal = c("loadings","intercepts","residuals"), estimator = "WLSMV")

measEqOut <- compareFit(fit.config, fit.metric, fit.scalar, fit.strict)

summary(measEqOut)

# Further validation

#install.packages("QuantPsyc", dependencies = TRUE)

library("Hmisc")

subdat <- subset(datas, select = c(Gender_r, Religiosity,Individualizing,Care,Eqauality,Binding,Proportionality,Loyalty,Authority,Purity))

res2 <- rcorr(as.matrix(subdat ))

res2

library(psych)

describeBy(datas, datas$Gender_r)

t.test(Individualizing ~ Gender_r, data = datas, var.equal=T, alternative= 'two.sided')

t.test(Care ~ Gender_r, data = datas, var.equal=T, alternative= 'two.sided')

t.test(Eqauality ~ Gender_r, data = datas, var.equal=T, alternative= 'two.sided')

t.test(Binding ~ Gender_r, data = datas, var.equal=T, alternative= 'two.sided')

t.test(Proportionality~ Gender_r, data = datas, var.equal=T, alternative= 'two.sided')

t.test(Loyalty~ Gender_r, data = datas, var.equal=T, alternative= 'two.sided')

t.test(Authority ~ Gender_r, data = datas, var.equal=T, alternative= 'two.sided')

t.test(Purity ~ Gender_r, data = datas, var.equal=T, alternative= 'two.sided')

# install.packages("lsr", dependencies = TRUE)

library(lsr)

cohensD(datas$Individualizing ~ datas$Gender_r)

cohensD(datas$Care ~ datas$Gender_r)

cohensD(datas$Eqauality ~ datas$Gender_r)

cohensD(datas$Binding ~ datas$Gender_r)

cohensD(datas$Proportionality ~ datas$Gender_r)

cohensD(datas$Loyalty ~ datas$Gender_r)

cohensD(datas$Authority ~ datas$Gender_r)

cohensD(datas$Purity ~ datas$Gender_r)

mult.reg <- lm(datas$Religiosity ~ datas$Individualizing + datas$Binding)

summary(mult.reg)

library(car)

vif(mult.reg)

library(QuantPsyc)

stdcoeff <- lm.beta(mult.reg)

stdcoeff

# Cross-validation RMSE Root Mean Squared Error

#install.packages("Metrics", dependencies = TRUE)

library(caret)

library(ModelMetrics)

library(Metrics)

# Create 5 folds

folds <- createFolds(datas$Religiosity , k = 5)

# Initialize a vector to store RMSE values

rmse_values <- numeric(5)

set.seed(123)

# Perform cross-validation

for (i in 1:5) {

# Split data into training and validation sets

train_data <- datas[-folds[[i]], ]

valid_data <- datas[folds[[i]], ]

# Train the model

model <- lm(Religiosity ~Individualizing+Binding, data = train_data)

# Make predictions on validation set

predictions <- predict(model, newdata = valid_data)

# Calculate RMSE

rmse_values[i] <- rmse(valid_data$Religiosity , predictions)

}

# Calculate average RMSE

mean_rmse <- mean(rmse_values)

sd_rmse <- sd(rmse_values)

print(paste("Average RMSE:", mean_rmse, sd_rmse))

# Cross-validation Mean Absolute Error (MAE)

set.seed(123)

# Configuration of cross validation

ctrl <- trainControl(method = "cv", number = 5,

summaryFunction = defaultSummary)

# Training of the model and validation

model <- train(Religiosity ~ Individualizing+Binding, data = datas,

method = "lm", # oder eine andere Methode

trControl = ctrl,

metric = "MAE")

# MAE for every Fold

fold_results <- model$resample

# Avarage MAE for all Folds

mean_mae <- mean(fold_results$MAE)

sd_mae <- sd(fold_results$MAE)

print(paste("Durchschnittlicher MAE:", mean_mae, sd_mae))

# Cross-Validation r^2

library(caret)

set.seed(123)

# Perform cross-validation

cv_model <- train(Religiosity ~ Individualizing+Binding, data = datas, method = "lm",

trControl = trainControl(method = "cv", number = 5))

# extract R-squared values

r_squared_values <- cv_model$resample$Rsquared

# calculate the mean R squared value

mean_r_squared <- mean(r_squared_values)

# Result output

print(paste("Mittlerer R²-Wert:", round(mean_r_squared, 4)))

# Analyses for the six fundaments

mult.reg2 <- lm(datas$Religiosity ~ datas$Care + datas$Eqauality + datas$Proportionality+datas$Loyalty+datas$Authority+datas$Purity)

summary(mult.reg2)

vif(mult.reg2)

library(QuantPsyc)

stdcoeff <- lm.beta(mult.reg2)

stdcoeff

# Cross-validation Root Mean Squared Error (RMSE)

#install.packages("Metrics", dependencies = TRUE)

library(caret)

library(ModelMetrics)

library(Metrics)

# Create 5 folds

folds <- createFolds(datas$Religiosity , k = 5)

# Initialize a vector to store RMSE values

rmse_values <- numeric(5)

set.seed(123)

# Perform cross-validation

for (i in 1:5) {

# Split data into training and validation sets

train_data <- datas[-folds[[i]], ]

valid_data <- datas[folds[[i]], ]

# Train the model

model <- lm(Religiosity ~ Care + Eqauality + Proportionality+ Loyalty+ Authority + Purity, data = train_data)

# Make predictions on validation set

predictions <- predict(model, newdata = valid_data)

# Calculate RMSE

rmse_values[i] <- rmse(valid_data$Religiosity , predictions)

}

# Calculate average RMSE

mean_rmse <- mean(rmse_values)

sd_rmse <- sd(rmse_values)

print(paste("Average RMSE:", mean_rmse, sd_rmse))

# Cross-Validated Mean Absolute Error (MAE)

set.seed(123)

# configuring cross-validation

ctrl <- trainControl(method = "cv", number = 5,

summaryFunction = defaultSummary)

# train and validate model

model <- train(Religiosity ~ Care + Eqauality + Proportionality+Loyalty+Authority+Purity, data = datas,

method = "lm", # oder eine andere Methode

trControl = ctrl,

metric = "MAE")

# MAE for every Fold

fold_results <- model$resample

# Average MAE for all Folds

mean_mae <- mean(fold_results$MAE)

sd_mae <- sd(fold_results$MAE)

print(paste("Durchschnittlicher MAE:", mean_mae, sd_mae))

# Cross-validated R^2

library(caret)

set.seed(123)

# Perform cross-validation

cv_model <- train(Religiosity ~ datas$Care + datas$Eqauality + datas$Proportionality+datas$Loyalty+datas$Authority+datas$Purity, data = datas, method = "lm",

trControl = trainControl(method = "cv", number = 5))

# Extract R-squared values

r_squared_values <- cv_model$resample$Rsquared

# Calculate the average R² value

mean_r_squared <- mean(r_squared_values)

# print results

print(paste("Mittlerer R²-Wert:", round(mean_r_squared, 4)))

# Only care and equality

mult.reg2 <- lm(datas$Religiosity ~ datas$Care + datas$Eqauality )

summary(mult.reg2)

vif(mult.reg2)

library(QuantPsyc)

stdcoeff <- lm.beta(mult.reg2)

stdcoeff

# Cross-validation RMSE Root Mean Squared Error

#install.packages("Metrics", dependencies = TRUE)

library(caret)

library(ModelMetrics)

library(Metrics)

# Create 5 folds

folds <- createFolds(datas$Religiosity , k = 5)

# Initialize a vector to store RMSE values

rmse_values <- numeric(5)

set.seed(123)

# Perform cross-validation

for (i in 1:5) {

# Split data into training and validation sets

train_data <- datas[-folds[[i]], ]

valid_data <- datas[folds[[i]], ]

# Train the model

model <- lm(Religiosity ~ Care + Eqauality , data = train_data)

# Make predictions on validation set

predictions <- predict(model, newdata = valid_data)

# Calculate RMSE

rmse_values[i] <- rmse(valid_data$Religiosity , predictions)

}

# Calculate average RMSE

mean_rmse <- mean(rmse_values)

sd_rmse <- sd(rmse_values)

print(paste("Average RMSE:", mean_rmse, sd_rmse))

# Cross-validation Mean Absolute Error (MAE)

set.seed(123)

# Cross-validation configuration

ctrl <- trainControl(method = "cv", number = 5,

summaryFunction = defaultSummary)

# Train and validate model

model <- train(Religiosity ~ Care + Eqauality, data = datas,

method = "lm", # oder eine andere Methode

trControl = ctrl,

metric = "MAE")

# MAE for every Fold

fold_results <- model$resample

# Average MAE across all folds

mean_mae <- mean(fold_results$MAE)

sd_mae <- sd(fold_results$MAE)

print(paste("Durchschnittlicher MAE:", mean_mae, sd_mae))

# Cross-validation r^2

library(caret)

set.seed(123)

# Perform cross-validation

cv_model <- train(Religiosity ~ datas$Care + datas$Eqauality, data = datas, method = "lm",

trControl = trainControl(method = "cv", number = 5))

# Extract R² values

r_squared_values <- cv_model$resample$Rsquared

# Calculate the average R² value

mean_r_squared <- mean(r_squared_values)

# Print Results

print(paste("Mittlerer R²-Wert:", round(mean_r_squared, 4)))
